# Body Evaluation and Body Ownership in Patients with Inflammatory Bowel Disease: the Role of Interoceptive Sensibility and Childhood Maltreatment

**DOI:** 10.1007/s12529-024-10316-z

**Published:** 2024-08-21

**Authors:** Konstantina Atanasova, Tobias Lotter, Robin Bekrater-Bodmann, Nikolaus Kleindienst, Anne Kerstin Thomann, Stefanie Lis, Wolfgang Reindl

**Affiliations:** 1 Department of Medicine II, Medical Faculty Mannheim, Universitätsklinikum Mannheim, Heidelberg University, Theodor-Kutzer-Ufer 1-3, Haus 8, Ebene 4, 68167 Mannheim, Germany; 2 Department of Clinical Psychology, Medical Faculty Mannheim, Central Institute of Mental Health, Heidelberg University, Mannheim, Germany; 3 Department of Psychosomatic Medicine and Psychotherapy, Center for Internal Medicine, University Medical Center Hamburg-Eppendorf, Hamburg, Germany; 4 Department of Psychiatry, Psychotherapy and Psychosomatics, Faculty of Medicine, RWTH Aachen, Aachen, Germany; 5 Department of Psychosomatic Medicine, Medical Faculty Mannheim, Central Institute for Mental Health Mannheim, Heidelberg University, Mannheim, Germany

**Keywords:** Body evaluation, Body ownership, Childhood trauma, Childhood maltreatment, Inflammatory bowel disease, Interoception

## Abstract

**Objective:**

Inflammatory bowel diseases (IBD) are accompanied by symptoms that can vastly affect patients’ representations of their bodies. The aim of this study was to investigate alterations in body evaluation and body ownership in IBD and their link to interoceptive sensibility, gastrointestinal-specific anxiety, and history of childhood maltreatment.

**Methods:**

Body evaluation and ownership was assessed in 41 clinically remitted patients with IBD and 44 healthy controls (HC) using a topographical self-report method. Interoceptive sensibility, gastrointestinal-specific anxiety and a history of childhood maltreatment were assessed via self-report questionnaires.

**Results:**

Patients reporting higher interoceptive sensibility perceived their bodies in a more positive manner. Higher gastrointestinal-specific anxiety was linked to a more negative body evaluation particularly of the abdomen in patients with IBD. Childhood maltreatment severity strengthened the positive association between interoceptive sensibility and body ownership only in those patients reporting higher trauma load.

**Conclusion:**

Altered body representations of areas associated with abdominal pain are linked to higher symptom-specific anxiety and lower levels of interoceptive sensibility in IBD. Particularly in patients with a history of childhood maltreatment, higher levels of interoceptive sensibility might have a beneficial effect on the patients’ sense of body ownership.

**Supplementary Information:**

The online version contains supplementary material available at 10.1007/s12529-024-10316-z.

## Introduction

Inflammatory bowel diseases (IBD) are chronic immune-mediated inflammatory conditions of the gastrointestinal tract, hallmarked by severe symptoms known to negatively affect how patients perceive their bodies [1]. From a clinical point of view, two facets of body representations are of particular interest in IBD: body evaluation, that is, an individual’s attitude towards their own body [2], and body ownership, the sense of that the body is belonging to oneself [3]. Several studies have demonstrated dysfunctional body-related attitudes in patients with IBD, characterized by lower levels of body satisfaction [1, 4–6]. Moreover, IBD symptoms can be experienced as an attack originating from within the body, affecting a patient’s experience of the body as their own [7]. To date, little is known about the role of specific psychological factors in those facets of body representations in IBD.

Contemporary findings have emphasized the important role of interoceptive processes in how individuals perceive their bodies. These have indicated poorer interoceptive abilities to be associated with decreased levels of body satisfaction [8–10]. While persons with IBD do not differ in their objective ability to perceive bodily sensations (interoceptive accuracy), they appraise these differently (interoceptive sensibility) compared to unaffected individuals [11]. Furthermore, persons with IBD reporting greater levels of gastrointestinal-specific anxiety (GSA) experience disease-related bodily sensations as more distressing [12], which can aggravate disturbances in their body representations. Although no study has investigated the role of interoceptive processes in how patients with IBD perceive their bodies, it is conceivable that alterations in patients’ interoceptive sensibility and higher GSA might contribute to the development and maintenance of body representation disturbances during the disease course.

The sense of body ownership arises from the multisensory integration of interoceptive and exteroceptive cues. For that reason, the extent to which an individual perceives their body as belonging to themselves depends on the sensitivity to their bodily signals [13–15]. While some previous evidence has indicated impairments in the multisensory integration in patients with immune-mediated diseases, resulting in a disturbed sense of body ownership [7], this has not been investigated in IBD populations yet. On the one hand, as persons with IBD tend to pay greater attention to their disease-related visceral sensations [16], this might be linked to a stronger perception of the body as “their own” as it is the “own” body that experiences these symptoms. However, as body ownership depends on the constant flow of sensory input, the occurrence of pain symptoms over a prolonged period of time may promote alterations in the integration of interoceptive signals, resulting in a disturbed sense of ownership for the affected parts of the body [17–19]. Some empirical evidence has suggested that body parts affected by symptoms of complex regional pain syndrome are perceived as less belonging to oneself [20, 21]. Thus, in IBD, body areas associated with abdominal pain might be linked to a decreased body ownership of these parts of the body.

Body representation disturbances have been repeatedly observed not only in medical conditions [7, 22, 23] but also in several mental health conditions [24–26]. The term ‘childhood trauma’ encompasses a spectrum of maltreatments occurring during early life. This includes emotional and physical abuse or neglect as well as sexual abuse. Early life exposure to trauma is often of an interpersonal nature, includes violation of one ‘s physical integrity and occurs during vulnerable periods of child’s development, often resulting in symptoms of post-traumatic stress disorder (PTSD) such as avoidance or numbing [27–29]. An often overlooked topic in the field of trauma research is the long-lasting negative impact of childhood trauma on individual’s relationship with their body [30]. Previous studies have demonstrated that individuals reporting childhood maltreatment such as sexual abuse often experience difficulties in perceiving their bodies as their own [31, 32]. The experience of the body as „my body “ develops in early life and is based on physical experiences and explicit definitions of boundaries between the self and others [33, 34]. In case of physical and sexual maltreatment, memories of these traumatic experiences are often body-related and may result in rejection of one’s own body [35], a loss of contact with it [25, 36], and the experience of threatened physical integrity [37]. As a result, individuals exposed to childhood trauma often experience difficulties in perceiving inner sensations and tend to deny interoceptive awareness [30, 38]. Individuals exposed to childhood trauma were shown to exhibit higher risk of revictimization later in life (for review: [39]). Lifetime exposure to traumatic events can negatively affect an individual’s body perception, being associated with symptoms of post-traumatic stress such as catastrophic and frightful orientation towards bodily signals [40].

Childhood maltreatment has been shown to have severe negative consequences for individual’s physical health as well. Persons exposed to childhood trauma have elevated levels of inflammatory markers as adults and exhibit greater immune reactivity to stress compared to individuals without a history of traumatization [41, 42]. As childhood trauma can result in a chronic inflammatory state through an altered function of the hypothalamic–pituitary–adrenal (HPA) axis, it may predispose individuals to the development of autoimmune diseases. Indeed, immune-mediated inflammatory conditions such as IBD, multiple sclerosis, and rheumatoid arthritis have been shown to be associated with a higher prevalence of childhood maltreatment [43, 44]. However, the links between traumatic experiences during childhood and body evaluation or body ownership have not yet been evaluated in IBD populations.

The aim of the present study was to examine the complex interplay of body representations, interoceptive sensibility, GSA, and a history of childhood maltreatment in IBD. We hypothesized that compared to healthy individuals, patients with IBD (I.) report a more negative body evaluation and (II.) a more negative body evaluation is associated with higher GSA, lower interoceptive sensibility, lower self-esteem, and a history of childhood maltreatment. Furthermore, we expected the IBD group to (III.) report greater levels of whole-body ownership due to patients’ increased attention towards their bodily sensations and that experience of pain is associated with altered sense of ownership for the affected body parts. We expected that (IV.) higher interoceptive sensibility in IBD is linked to stronger whole-body ownership. Finally, we hypothesized that (V.) a history of childhood maltreatment is negatively associated with body ownership and (VI.) moderates the association between body ownership and interoceptive sensibility in IBD. This hypothesis was based on previous findings indicating a positive association between interoception and the sense of ownership [14], while a history of traumatization is known to negatively affect the perception of bodily signals [45] and body representations [25].

## Materials and Methods

### Participants

The study was approved by the Ethics Committee of the Medical Faculty Mannheim at Heidelberg University and all participants gave their written informed consent before participating in the study. An a priori power analysis was conducted using G*Power3.1 [46] to define the required sample size for detecting between-group effects given an effect size of f = 0.25, α = 0.05 and 80% power. This analysis revealed that a total sample of 82 participants with two equal sized groups of n = 41 was required. In total, 86 individuals (age 18—65 years) participated in the study. Of these, 42 had a diagnosis of IBD and 44 were healthy control participants (HC). IBD diagnosis was based on endoscopic/histological and clinical findings [47–50]. We excluded one participant of the clinical group, due to insufficient comprehension of the experimental procedure. The final sample consisted of 41 IBD patients (20 female, 21 male) and 44 HC (30 female, 14 male). IBD outpatients were screened from the IBD outpatient unit at Department of Medicine II., University Medical Centre Mannheim during the routinely control visits and invited to participate in the study. Patients, who met the inclusion criteria requirements and provided written informed consent, took part in the study after the physical examination. Overall, 29 patients with Crohn’s disease (CD) and 12 patients with ulcerative colitis (UC) in clinical remission (Harvey-Bradshaw Index < 5; Partial Mayo Score ≤ 3; faecal calprotectin < 200 mg/kg; C-reactive protein < 5 mg/L) were included in the IBD group. Details on treatment medication, disease duration, symptoms severity, extraintestinal manifestations, history of further somatic diseases, and disease-related surgeries in the IBD group are provided in Tables S1 and S2, Supplementary material. We focused on patients with IBD in clinical remission to avoid potentially confounding effects of symptoms of active disease on the body evaluation and body ownership measures. Diagnostic procedures and gastroenterological examinations were carried out on all patients by fully trained physicians specialized in the care of patients with IBD. Exclusion criteria were biological signs of disease activity, current use of corticosteroids, use of psychotropic medications, and current or past neurological or psychiatric diseases. For further details on sample characteristics, see Table 1. Exclusion criteria for the HC group were: chronic medical conditions, use of psychotropic medication, current or past neurological or psychiatric diseases, and general gastrointestinal complaints during the last 4 weeks prior to the experiment. All HC were screened with a short structured clinical interview for psychiatric disorders (Mini-DIPS, Margraf and Cwik [51]). A trained clinical psychologist who was familiar with the DSM-5 classification and diagnostic criteria performed telephone interviews with all HC. Gastrointestinal complaints in the HC group were assessed during the telephone interview, assessing the occurrence, frequency, and severity of abdominal pain, diarrhea or constipation, abdominal cramps, and nausea in the last 4 weeks. All participants answered a set of psychometric questionnaires assessing GSA, interoceptive sensibility, self-esteem, and histories of childhood maltreatment and traumatic experiences. After completing these, participants performed a computer-based task, assessing their body evaluation, body ownership, and the experience of pain in the body.

**Table 1:** Sample characteristics

	HC (M ± SD) *N* = 44		IBD (M ± SD) *N* = 41		Test-statistics		*p*-value	
Demographics								
Age	36.11	± 12.29	40.20	± 13.87		1.44 ^a^	.154	
Gender (female/male/diverse)	30/14/-		20/21/-			3.30 ^b^	.069	
BMI	24.16	± 4.72	26.65	± 6.46		1.99 ^a^	.050	*
Years of education	12.84	± 2.40	12.32	± 3.01		-0.89 ^a^	.376	
Interoceptive sensibility								
MAIA	2.57	± 0.54	2.58	± 0.57		0.70^a^	.944	
Visceral sensitivity								
VSI	-	-	27.42	± 15.28		-	-	
Self-esteem								
RSES	34.86	± 4.33	33.54	± 5.26		-1.23^a^	.222	
Childhood maltreatment								
CTQ total	36.06	± 7.00	35.73	± 10.77		-0.15 ^c^	.881	
Emotional abuse	8.51	± 2.96	8.18	± 3.73		-0.41 ^c^	.684	
Physical abuse	5.80	± 1.60	6.27	± 2.53		0.92 ^c^	.362	
Sexual abuse	5.14	± 0.55	5.55	± 2.00		1.12 ^c^	.272	
Emotional neglect	9.63	± 3.29	8.91	± 3.79		-0.84 ^c^	.405	
Physical neglect	6.97	± 1.71	6.81	± 2.34		-0.31 ^c^	.758	
Lifetime traumatic experiences								
LEC-5	1.11	± 1.30	1.52	± 1.56		1.15 ^c^	.254	
Post-traumatic stress symptoms								
PCL-5 total	5.54	± 5.94	15.47	± 12.37		4.13 ^c^	< .001	***
Cluster B	1.43	± 1.95	4.25	± 3.69		3.86 ^c^	< .001	***
Cluster C	0.74	± 1.20	1.81	± 2.13		2.50 ^c^	< .013	*
Cluster D	1.86	± 2.79	4.16	± 3.55		2.96 ^c^	.004	**
Cluster E	1.43	± 1.70	4.84	± 4.52		4.02 ^c^	< .001	***

*IBD* inflammatory bowel diseases group, *HC* healthy controls group, *BMI* body-mass index, *MAIA* multidimensional assessment of interoceptive awareness, *VSI* visceral sensitivity index, *RSES* rosenberg self-esteem scale, *CTQ* childhood trauma questionnaire, *LEC-5* life events checklist, *PCL-5* post-traumatic stress disorder checklist for DSM-V, Cluster B = intrusion symptoms, Cluster C = avoidance, Cluster D = negative alterations in cognitions and mood, Cluster E = alterations in arousal and reactivity, * p < .05, ** *p* < .01, *** p < .001, ^a^ t-value, ^b^ Chi^2, c^ Mann–Whitney U

### Questionnaires

#### Gastrointestinal-Specific Anxiety

Gastrointestinal-specific anxiety (GSA) was measured with the Visceral Sensitivity Index (VSI; Labus, Bolus [52]). The 15-item scale assesses worry, fear, vigilance, sensitivity, and avoidance as well as gastrointestinal-related cognitions and behaviours (e.g. “When I feel discomfort in my belly, it frightens me”). Items are scored on a reversed 6-point scale with an overall VSI score (range: 0–75), with higher scores indicating more severe GSA. VSI was developed specifically for patients with functional gastrointestinal disorders, which is why we used this self-report measure in the IBD group only. Cronbach’s α of this scale for the current IBD sample was 0.89.

#### Interoceptive Sensibility

Interoceptive sensibility was assessed using the Multidimensional Assessment of Interoceptive Awareness (MAIA; Mehling, Price [53]). The 32-item self-report questionnaire provides a multidimensional profile of interoceptive sensibility, including the following eight subscales: Noticing, Not-Distracting, Not-Worrying, Attention Regulation, Emotional Awareness, Self-Regulation, Body Listening, and Trusting (e.g., “When I am tense, I notice where the tension is located in my body”; subscale range: 0–5). Higher scores indicate higher interoceptive sensibility. As we did not have specific hypotheses regarding the link between these different aspects of interoceptive sensibility and body representations, the mean MAIA total score (range: 0–40) was used for statistical analyses. Cronbach’s α was 0.91 in the IBD group and 0.94 in the HC group.

#### Self-Esteem

Self-esteem was assessed using the Rosenberg Self-Esteem Scale (RSES; Rosenberg [54]). The 10-item self-report questionnaire measures general self-esteem on a 4-point Likert scale, with higher scores indicating greater self-esteem (e.g., “I certainly feel useless at times”; range: 0–30). Cronbach’s α in the current sample was 0.86 in the IBD group and 0.85 in the HC group.

#### Childhood Trauma

The German version of the Childhood Trauma Questionnaire (CTQ; Klinitzke, Romppel [55]) was used to assess the history of childhood maltreatment. The 28-item questionnaire consists of five subscales including *emotional abuse* (e.g., “I felt that someone in my family hated me”), *physical abuse* (e.g., “I was punished with a belt, a board, a cord, or some other hard object”), *sexual abuse* (e.g., “Someone molested me”), *emotional neglect* (e.g., “People in my family felt close to each other”), and *physical neglect* (e.g., “I didn’t have enough to eat”) (subscale range: 0–25) as well as a total score (range: 0–125). Higher CTQ scores indicate a more severe history of childhood maltreatment. CTQ revealed acceptable to good internal consistency with Cronbach’s α = 0.86 in the IBD group and 0.76 in the HC group.

#### Lifetime Traumatic Experiences and Post-traumatic Stress Symptoms

All participants were screened for potentially traumatic life events using the Life Events Checklist for DSM-5 (LEC-5; Weathers, Litz [56]), a self-report measure assessing the exposure to 16 events known to potentially lead to a PTSD. For each participant, the number of personally experienced traumatic events (direct exposure) was summed up, resulting in a single sum score (range: 0–16). Severity of PTSD symptoms was evaluated with the PTSD Checklist for DSM-5 (PCL-5; Weathers, Litz [56]), that is, a 20-item self-report measure assessing the presence and severity of posttraumatic stress (e.g., “In the past month, how much were you bothered by repeated, disturbing dreams of the stressful experience?”; range: 0–80). Higher scores indicate higher PTSD symptoms severity. PCL-5 revealed high internal consistency in the current study with Cronbach’s α = 0.91 in the IBD group and 0.87 in the HC group.

### Experimental Procedure and Dependent Variables

Participants were asked to appraise their body evaluation and body ownership using a topographical self-report method adapted from Nummenmaa et al. [57] and customized for the purposes of this study. In the original version of this tool, participants were shown silhouettes of a human body and were asked to indicate the location where activity changes in their body occur in association with a given emotional state. In contrast to the tool of Nummenmaa, Glerean [57], no emotional states were given in the present study. Instead, participants were familiarized with the concepts of body evaluation and body ownership at the beginning of the task and were asked to indicate location and manifestation of the respective concept in the provided silhouettes. Similar to the original tool, participants could choose different colours from a pre-defined colour bar to indicate their response and paint the corresponding body areas by successive strokes or continuous painting with their cursor, controlled by a computer mouse. Participants evaluated to what extent they like their body parts and to what extent they perceive their body parts as belong to themselves on a 9-point colour bar ranging from blue (-4 = “very much dislike”; “does not belong to myself”) to red (+ 4 = “very much like”; “belongs to myself”). In two additional trials, participants’ pain experience at the time of the experiment and during the preceding four weeks was assessed using a 5-point colour bar corresponding to the different intensities of the perceived pain (0 = “no pain at all” to 4 = “very strong pain”). All four instructions were presented in a pseudorandomized order. The task was presented on a 14’’ computer screen. Front and back body templates comprised 366 × 195 pixels and the diameter of the painting tool was 13 pixels. The task was programmed using Presentation® software (Version 20.1, Neurobehavioral Systems, Inc., Berkeley, CA, www.neurobs.com). For each participant, a whole-body score was calculated as well as scores for the regions of interest (ROI) defined by the presence of pain experiences evaluated across all participants. Computations were done after applying a spatial smoothing of 3 pixels at a single-subject level by averaging the colour code value of each coloured pixel and its surrounding pixels.

#### Whole-body Scores

Whole-body scores were averaged separately for each participant for body evaluation and body ownership ratings across the colour codes of all pixels of the presented body template, which were coloured by the participant.

#### Pain-Related ROI

**Fig. 1:**
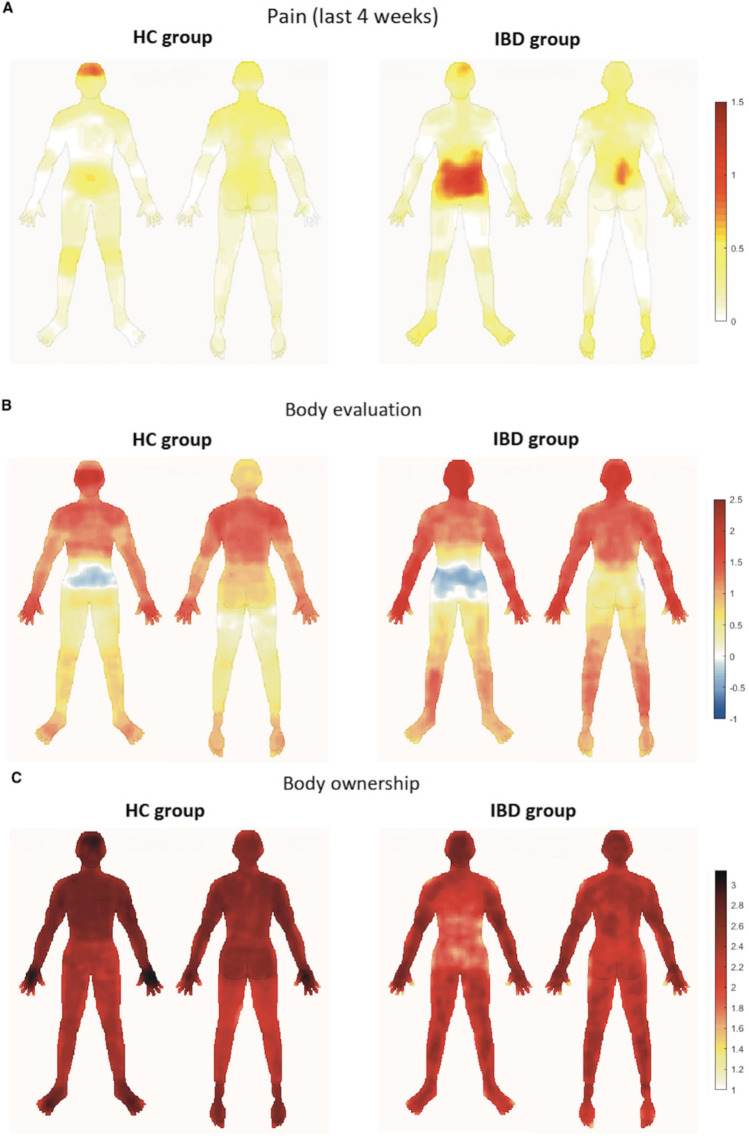
Grand averages of the body topographies for (**A**) reported pain experience during the last four weeks preceding the experiment (0/white = “no pain at all”; 4/red = “very strong pain”), **B** body evaluation (-4/blue = “very much dislike”; 4/red = “very much like”), and (**C**) body ownership (-4/blue = “does not belong to myself”; 4/red = “belongs to myself”). Note: for illustration purposes the colour bars have been adjusted differentially to the range of values for the different instructions

Based on the reported mean pain ratings across all participants (M = 1.61, SD = 0.74; see Supplementary material, Figure S1), pain-related ROI were defined as pixels with a mean pain intensity ≥ 1.60 during the preceding four weeks (Fig. 1A). Three distinct body areas were identified: head (ROI size: 757 pixels), abdomen (2227 pixels), and back (877 pixels) (see Supplementary material, Figure S2). Since we aimed to investigate IBD-specific alterations in body representations, we decided to differentiate between areas related to abdominal pain (abdomen area) and areas related to non-abdominal pain perception (head and back area) ROI. For these two ROI clusters, intensity scores were averaged separately for each participant and condition. Whole-body and ROI analyses were performed using custom pipelines run with MATLAB 2020a (The MathWorks, Inc.).

### Data Analysis

Shapiro–Wilk tests and the corresponding Q-Q plots indicated that the observed data was positively skewed and violated the normal distribution assumptions. Thus, nonparametric tests were implemented to analyse group differences. Whole-body and ROI scores were compared between both groups using Mann–Whitney U tests separately for body evaluation, body ownership, and pain ratings. To analyse covariation between body evaluation and body ownership scores and participants’ interoceptive sensibility, history of childhood trauma, and self-esteem, Spearman’s rank correlation coefficients were computed. Additionally, associations between body evaluation, body ownership, interoception, and GSA in the IBD group were analysed using Spearman’s rank correlation coefficients. To test severity of childhood maltreatment as potential moderator of the association between body ownership and interoceptive sensibility, a moderation analysis with bootstrapping (5,000 samples with replacement) was applied (PROCESS SPSS macro version 3.5.2) [58]. Since the observed variance within the different types of maltreatment was rather low, we used the CTQ total scores instead of the subscale scores to perform these moderation analyses.

Statistical analyses were carried out with SPSS v.27.0 (IBM Corp., USA). For all analyses, statistical significance was set to *p* < 0.05. The level of confidence for all reported confidence intervals was set to 95%. To control for multiple testing, we applied a false-discovery rate (FDR) correction according to Benjamini & Hochberg [59] and reported the corresponding *p*-values by the subscript *p*_*FDR*_. For whole-body scores, FDR correction was applied for the number of instructions being compared. For group comparisons of the ROI scores within each instruction, *p*-values were adjusted according to the number of ROI.

## Results

### Whole-body and ROI Analyses

For illustrative purposes, grand averages of the reported ratings are displayed for each group and condition with body silhouettes as overlays (Fig. 1). Whole-body analyses revealed no differences between patients and HC either for their pain experience during the experiment (U (n _IBD_ = 41, n _HC_ = 44) = 787.00, *p* = 0.30, *p*_*FDR*_ = 0.60) or the preceding four weeks (U = 855.50, *p* = 0.83, *p*_*FDR*_ = 0.83), their body evaluation (U = 807.50, *p* = 0.91, *p*_*FDR*_ = 0.91) or body ownership (U = 560.50, *p* = 0.09, *p*_*FDR*_ = 0.19). ROI analyses revealed stronger pain experience in the abdomen in the IBD group compared to HC during task solving (U = 622.00, *p* =0.001*, p*_*FDR*_ = 0.002) as well as during the last four weeks (U = 550.00, *p* = 0.001, *p*_*FDR*_ = 0.002). While both groups did not differ in their evaluation of this body area (U = 666.50, *p* = 0.86, *p*_*FDR*_ = 0.86), patients experienced the abdomen as significantly less belonging to themselves compared with HC (U = 453.00, *p* = 0.01, *p*_*FDR*_ = 0.02). No significant group differences were found for the remaining ROI (head, back) (all *p*_*FDR*_ ≥ 0.53; see Table 2). The groups did not differ in the number of coloured pixels either for body evaluation or body ownership trials (for more information see Supplementary material, Table S3).

**Table 2:** Group differences in pain experience, body evaluation and body ownership

*N* =	HC (M ± SD) *N* = 41		IBD (M ± SD) *N* = 44		Mann–Whitney U		*p*-value *p p*_*FDR*_		
*N* =	1.62	± 0.84	1.61	± 0.64	855.50	.826		.826	
*N* =	0.65	± 0.69	0.84	± 0.81	787.00	.299		.598	
*N* =	**0.49**	** ± 0.92**	**1.18**	** ± 1.10**	**550.00**	**.001**		**.002**	**
*N* =	0.49	± 0.92	0.72	± 0.80	884.50	.875		.875	
*N* =	**0.08**	** ± 0.32**	**0.56**	** ± 0.87**	**622.00**	**.001**		**.002**	**
*N* =	0.14	± 0.30	0.19	± 0.42	878.00	.772		.875	
*N* =									
*N* =	1.16	± 1.61	1.08	± 1.74	807.50	.913		.913	
*N* =	0.01	± 2.43	-0.14	± 2.36	666.00	.862		.862	
*N* =	1.49	± 1.55	1.59	± 1.69	641.00	.791		.791	
*N* =									
*N* =	3.13	± 1.07	2.90	± 0.95	560.50	.094		.188	
*N* =	**3.08**	** ± 1.58**	**2.25**	** ± 1.98**	**453.00**	**.012**		**.024**	*
*N* =	3.07	± 1.23	2.97	± 0.92	577.50	.186		.372	

*IBD* inflammatory bowel disease group, *HC*, healthy control group, *ROI*, region of interest, *FDR* false discovery rate; *p < .05, **p < .01

### Correlation Analyses

#### Associations of Body Evaluation and Body Ownership with GSA

A more negative evaluation of the abdomen but not of the whole body or of the remaining ROI (all *p*_*FDR*_ ≥ 0.13) was related to higher GSA in the IBD group (*r*_*s*_ = -0.373, *p* = 0.03) (see Figure S3, Supplementary material). However, this association did not survive the correction for multiple comparisons (*p*_*FDR*_ = 0.07). Body ownership was not associated with GSA (all *p*_*FDR*_ ≥ 0.43).

#### Associations of Body Evaluation and Body Ownership with Interoceptive Sensibility

Across all participants, body evaluation was not related to interoceptive sensibility (*r*_*s*_ = 0.187, *p* = 0.11, *p*_*FDR*_ = 0.15). Separate analyses for both groups revealed that this link was significant in the IBD group (*r*_*s*_ = 0.344, *p* = 0.04, *p*_*FDR*_ = 0.04) but not in the HC group (*r*_*s*_ = -0.060, *p* = 0.71, *p*_*FDR*_ =0.92). A similar significant association was also found for the abdomen (*r*_*s*_ = 0.366, *p* = 0.03, *p*_*FDR*_ = 0.04) as well as the remaining ROI scores (*r*_*s*_ = 0.450, *p* = 0.007, *p*_*FDR*_ = 0.021) among the patients, indicating that a more positive evaluation of these body areas was associated with higher interoceptive sensibility. Interoceptive sensibility was not significantly linked to body ownership either across all participants (all *p*_*FDR*_ ≥ 0.19) or in IBD or HC participants (all *p*_*FDR*_ ≥ 0.55).

#### Associations of Body Evaluation and Body Ownership with History of Childhood Trauma

Whole-body evaluation and ownership scores were not associated with history of ACE in IBD (*p*_*FDR*_ ≥ 0.32) nor in HC (*p*_*FDR*_ ≥ 0.94). After applying correction for multiple comparisons, no significant associations were found for the body evaluation scores of the ROI in IBD (all *p*_*FDR*_ ≥ 0.09) or HC (*p*_*FDR*_ ≥ 0.79). In line with our hypothesis, a more severe history of ACE was associated with lower ownership scores for the abdomen among IBD patients (*r*_*s*_ = -0.383, *p* = 0.02, *p*_*FDR*_ = 0.04) but not in HC (*r*_*s*_ = 0.077, *p* = 0.65, *p*_*FDR*_ = 0.65).

#### Moderation of the Associations of Body Ownership with Interoceptive Sensibility by Childhood Trauma

Moderation analyses across all participants revealed a significant moderation effect of trauma severity on the association between interoceptive sensibility and ownership scores for the abdomen area (*F*(1,70) = 4.62, B = 0.12, CI [0.009, 0.241], *p* = 0.04). The model explained 9% of the variance (*R*^*2*^ = 0.09; *F*(3,81) = 2.25, *p* = 0.09)). A significant interaction effect between interoceptive sensibility and trauma severity was found in the IBD group (*F*(1,33) = 4.76, B = 0.18, CI [0.012, 0.338], *p* = 0.04) but not in the HC group (*F*(1,33) = 0.21, B =—0.04, CI [- 0.226, 0.144], *p* = 0.65) (see Supplementary material, Figure S4). Further analyses within the IBD group revealed that the moderating effect of histories of childhood maltreatment was significant only for patients with moderate CTQ scores (B = 2.58, CI [0.074, 5.094], *p* = 0.04) but not for those without childhood trauma or low trauma severity (all *p* ≥ 0.22). Thus, trauma severity strengthened the positive association between interoceptive sensibility and body ownership scores only in those patients reporting higher trauma load (Supplementary material, Figure S4 A). Trauma severity did not moderate the link between interoceptive sensibility and the ownership scores for the whole-body or for the head and the back areas (all *p*s ≥ 0.21).

#### Associations Between Body Evaluation, Self-esteem, and BMI

Contrary to our hypothesis, across all participants self-esteem was not associated with body evaluation ratings either for the whole body or for the pre-defined ROI (*p*_*FDR*_ ≥ 0.09). However, separate analyses for both groups revealed that higher self-esteem was linked to a more positive evaluation of the abdomen in IBD (*r*_*s*_ = 0.531, *p* = 0.002, *p*_*FDR*_ = 0.004). This association was not found in HC (*r*_*s*_ = 0.006, *p* = 0.97, *p*_*FDR*_ = 0.97). BMI was not associated with body evaluation among the patients (all *p*_*FDR*_ ≥ 0.33) or HC participants (all *p*_*FDR*_ ≥ 0.31). Exploratory analyses revealed no gender differences regarding body evaluation either in IBD (*p* ≥ 0.20) or HC (*p* ≥ 0.22) or between both groups (females: *p* ≥ 0.58; males: *p* ≥ 0.77). In the IBD group, the number of previous disease-related surgeries was not associated with body evaluation scores (*p* ≥ . 46) (see Supplementary material, Table S4).

## Discussion

IBD-associated disease symptoms can vastly affect a patient’s perception and satisfaction with their bodies [5, 6, 60]. The aim of the present study was to investigate whether patients with IBD show alterations in the way they perceive their bodies with respect to how positive or negative they tend to evaluate their bodies and to what extent they perceive their body parts as belonging to themselves.

Our findings revealed no differences with regard to body evaluation between patients with IBD and HC. In IBD, a more negative body evaluation of the abdomen was associated with higher levels of gastrointestinal-specific anxiety (GSA) and lower self-esteem, while a more negative evaluation of the whole body was related to lower levels of interoceptive sensibility. With respect to body ownership, patients with IBD perceived their abdomen as significantly less belonging to themselves compared to the healthy individuals. This indicates that disturbances in the sense of ownership might especially be related to body areas associated with the experience of disease-specific pain symptoms. In contrast to body evaluation, body ownership did not show significant associations with GSA, interoceptive sensibility, or self-esteem. However, moderation analyses indicated that the link between body ownership and interoceptive sensibility was influenced by the reported severity of childhood maltreatment in IBD specifically for the perception of the abdomen area.

### Whole-body and ROI Scores

In contrast to our hypothesis, patients with IBD did not perceive their bodies more negatively compared to HC. Previous findings have suggested lower body satisfaction in patients with active disease compared to those in clinical remission, indicating a significant link between a negative body evaluation and disease severity [5]. Our findings indicate no alterations in the whole-body evaluation of remitted patients, suggesting that disease activity might crucially affect the patients’ perception of their bodies. As we did not observe a more negative body evaluation in this sample, the tendency towards lower body satisfaction reported in previous studies might reflect state-like effects, primarily associated with acute disease symptoms. One could speculate that disturbances in patients’ body evaluation might not be persistent throughout the course of the disease but rather related to the periods of flare-ups, which are typically accompanied by severe physical symptoms. It should be stressed that previous studies reporting a decreased body satisfaction in IBD did not include healthy comparison groups [1, 5, 6]. It is also conceivable that certain body areas, such as the stomach area, are associated with higher levels of dissatisfaction in healthy adults, which might be comparable with IBD patients in remission. Future studies are needed to examine the interplay of symptoms severity and body evaluation in IBD in larger samples including patients in remission as well as those with active disease and compare them to unaffected individuals of all genders and age groups.

We investigated one further facet of body representations in IBD – the sense of body ownership. In contrast to our hypothesis and previous findings in medical conditions [7, 61] and mental health conditions [25], patients with IBD did not report altered levels of whole-body ownership compared to healthy individuals. Previous empirical findings have indicated an altered multisensory integration in patients with autoimmune diseases such as Coeliac Disease, resulting in a reduced sense of body ownership [7]. However, this observation was based on the Rubber Hand Illusion [62], an experimental paradigm during which participants are induced to perceive ownership for an artificial body part by the application of conflicting multisensory input. Although previous authors suggested that the Rubber Hand Illusion and interviews on habitual whole-body ownership assess similar processes [25] this suggestion is unverified yet; it may be that differential sensory and/or cognitive processes are involved. It may also be the case that patients in the remitted stages of the disease experience less disturbances in their sense of body ownership compared to patients with an acute flare-up, similar to the construct of body satisfaction; this state-dependent effect on body ownership has been shown for other stress-related disorders recently [25]. However, patients reported significantly lower ownership ratings for the abdomen, that is, the body area most affected by gastrointestinal pain [63]. This finding is in line with previous studies showing that i.e., patients with regional pain syndrome, report a feeling of “foreignness” towards body parts affected by the symptoms of pain [64]. Neuroimaging studies have demonstrated that both body ownership and pain perception share common neural underpinnings, including the somatosensory cortex and the insular cortex [65, 66]. In IBD, the cortical thickness in these regions is changed and linked to the chronically increased afferent input from the gut to the brain due to relapsing mucosal inflammation [67, 68]. Therefore, future studies are needed to examine whether cortical changes associated with the processing of visceral pain in IBD might potentially contribute to a disturbed sensory integration [7, 13], promoting alterations in patients’ sense of ownership for body parts affected by these pain symptoms.

### Associations with GSA, Interoceptive Sensibility and Self-esteem

Our results indicated that higher GSA in IBD is associated with a more negative evaluation of the abdomen but not with a diminished sense of ownership for this body area. A recent study demonstrated lower satisfaction with the abdomen in young adolescents diagnosed with IBD too [1]. In line with this finding, our results suggest that greater anxiety and worries about those symptoms are linked to a more negative body perception and stronger body dissatisfaction might be associated with a greater gastrointestinal-specific anxiety in IBD. However, it should be mentioned that after applying a correction for multiple comparisons, this association was no longer significant. Therefore, it needs to be interpreted cautiously and replicated in future studies. Longitudinal studies are required to investigate the interplay between body perception and these symptoms to test for causal relationships and/or bidirectional interplay.

Further, a higher interoceptive sensibility was linked to a more positive body evaluation specifically for the IBD group. This link was limited only to body areas associated with the experience of pain, suggesting a more general relationship between body evaluation and interoceptive sensibility in IBD. This finding is in line with previous research showing that individuals who are more aware of their bodily sensations report more positive attitudes towards the own body [10]. Since this association was significant only in the IBD group, this might imply that interoceptive sensibility might potentially have beneficial effects for patients’ body evaluation. However, because of the correlational nature of this association, no conclusions can be drawn regarding the causality and the direction of this link. Previous studies investigating the effects of mind–body interventions in gastrointestinal disorders demonstrated that practicing systematic attention to one’s body signals can have positive effects on the reported disease-related anxiety and quality of life [69–71]. As a more negative body evaluation has been linked to a poorer quality of life and greater psychological distress [72], interoceptively-focused interventions might be of particular importance in IBD sub-populations exhibiting body evaluation disturbances.

Contrary to our hypothesis, higher interoceptive sensibility was not linked to a stronger sense of body ownership. Most of the previous studies suggesting an association between individuals’ interoceptive abilities and body ownership have investigated the interoceptive performance by experimental heartbeat detection or discrimination tasks [14, 73, 74]. Our results suggest that self-reported awareness of bodily signals and its changes is not associated with body ownership as measured by a topographical self-report method. This might indicate that objective performance, rather than subjective beliefs about one’s interoceptive abilities, influence the sense of body ownership. Future studies are asked to explore the association of these different aspects of interoception and their relation to body ownership by implementing self-report methods as well as experimental tasks (e.g., a combination of RHI and heartbeat tracking/detection tasks).

Finally, a positive association between patients’ self-esteem and evaluation of the abdomen was observed in the IBD group. This result suggests that a more negative attitude towards body areas associated with the experience of pain might detrimentally affect patients’ general self-esteem [75]. As this relationship is only of correlational nature, it is also conceivable that individuals reporting a diminished self-esteem perceive this body area more negatively. Thus, further studies are needed to examine the nature and causality of this association.

### Body Evaluation, Body Ownership and Childhood Trauma

A history of childhood maltreatment was not associated with whole-body evaluation or body ownership. One potential explanation for the lack of significant associations between these aspects of body representation and childhood trauma might be the restricted variance of CTQ scores as observed in our sample. Besides this limitation, it is also conceivable that certain types of maltreatment, such as sexual or physical abuse, might affect body representations to a greater extent, since these are more strongly related to the experience of threatened physical integrity. Since the subsample of participants reporting histories of sexual or physical abuse was too small to perform additional statistical analyses, future studies should investigate the effects of different maltreatment types on body perception in IBD.

We found that patients with IBD reporting a more severe childhood trauma perceived their abdomen as less belonging to themselves. Moreover, patients with a more severe history of maltreatment showed a diminished sense of body ownership compared to those with no or only mild severity. This finding is in line with previous studies showing body ownership disturbances in patients with trauma-related disorders [25]. However, our results revealed that in the group of patients reporting a more severe history of childhood maltreatment, increasing levels of interoceptive sensibility were linked to an increased sense of body ownership. One could speculate that, a stronger attention to interoceptive sensations may contribute to an improved integration of sensory input, resulting in a stronger sense of ownership. Lower levels of body ownership could be potentially balanced out through an improvement in patients’ awareness of their body signals, especially in patients with a history of childhood trauma. Based on these considerations, future studies should further examine the effects of childhood maltreatment on body awareness and their link to body representations in IBD. Although preliminary, our findings point towards the importance of taking history of childhood trauma into account for future psychological treatments in IBD.

### Limitations

Some limitations of the present study have to be mentioned. First, based on our results, we cannot draw conclusions whether body representation disturbances found in remitted patients are also similar in patients with active disease. Given previous findings, it is conceivable that patients with active disease might experience their bodies in a more negative manner, especially because of a severe discomfort and disease-related symptoms. As our analyses indicated that representations of pain-related body areas in remitted IBD are still most severely affected even in the absence of acute inflammation, future research is needed to examine body representations and their link to IBD symptoms severity longitudinally over the course of the disease. Second, while our findings indicate the importance of childhood maltreatment as a moderating factor in the interplay of interoception and body ownership, our sample reported only low to moderate severity of childhood trauma according to the cut-off scores established in the literature [55]. As contemporary studies suggest a strong association between inflammation and a history of maltreatment in immune-mediated diseases [44], it is conceivable that a history of childhood trauma might promote stronger inflammatory activity and the development of psychiatric comorbidities. Therefore, the exclusion of patients with active disease and mental health conditions might explain the lower prevalence of childhood maltreatment in the present sample. In contrast to previous studies investigating the link between interoception and the sense of ownership, we only implemented self-report measures to assess these constructs. However, as the objective performance on experimental tasks measuring body perception often does not correspond with individual’s self-reports (as has been shown for interoception; [76]), further studies are needed that combine self-report measures with experimental task to deepen our understanding of the mechanism underlying changes in body ownership in IBD. One further limitation of our study is the lack of assessment of gastrointestinal discomfort and IBD-related symptoms at the time of experiment in addition to the assessment of pain sensations. Finally, the present findings rely on a cross-sectional design of the study and thus, no causal relationships between the investigated constructs can be inferred.

## Conclusions

In conclusion, our results demonstrate that patients with IBD in clinical remission perceive body areas affected by abdominal pain differently than unaffected individuals. Higher anxiety related to gastrointestinal symptoms was found to be related to a more negative body evaluation in IBD, which indicates that a patient’s exaggerated appraisal of their body signals might negatively affect their body perception even in the absence of acute symptoms. This is the first study showing that patients with IBD experience body parts affected by abdominal pain as less belonging to themselves, and this association was stronger in individuals reporting a history of childhood maltreatment. As patients reporting childhood trauma might constitute an especially vulnerable population, it might be useful to screen patients for history of childhood maltreatment and potential body perception disturbances in order to provide them with proper psychological support throughout the course of the disease. As we included only patients with remitted IBD, our results emphasize the importance of focussing not only on reducing inflammation through the disease course, but also to consider the long-term effects of IBD on the patients’ body perception in the absence of ongoing inflammation. An interdisciplinary team might help recognizing patients being at higher risk of negative body perception and associated mental health conditions and might offer them appropriate psychological support such as cognitive-behavioral therapy or mindfulness-based interventions.

## Supplementary Information

Below is the link to the electronic supplementary material.

Supplementary file1 (DOCX 676 KB)

## Data Availability

The data that support the findings of this study are available from the corresponding author, K.A, upon reasonable request.

## References

[1] Cushman G, et al. Age, disease symptoms, and depression are associated with body image dissatisfaction in newly diagnosed pediatric inflammatory bowel disease. J Pediatr Gastroenterol Nutr. 2021;72(3):e57–62. 32925551 10.1097/MPG.0000000000002943 PMC7870553

[2] Cash TF. Body image: past, present, and future. Body Image. 2004;1(1):1–5. 18089136 10.1016/S1740-1445(03)00011-1

[3] Botvinick M. Probing the neural basis of body ownership. Science. 2004;305(5685):782–3. 15297651 10.1126/science.1101836

[4] Claytor JD, et al. Body image dissatisfaction among pediatric patients with inflammatory bowel disease. J Pediatr. 2020;223:68-72.e1. 32711754 10.1016/j.jpeds.2020.04.045

[5] McDermott E, et al. Body image dissatisfaction: clinical features, and psychosocial disability in inflammatory bowel disease. Inflamm Bowel Dis. 2015;21(2):353–60. 25569732 10.1097/MIB.0000000000000287

[6] Saha S, et al. Body image dissatisfaction in patients with inflammatory bowel disease. Inflamm Bowel Dis. 2015;21(2):345–52. 25569736 10.1097/MIB.0000000000000270 PMC4373552

[7] Finotti G, Costantini M. Multisensory body representation in autoimmune diseases. Sci Rep. 2016;6(1): 21074. 26867786 10.1038/srep21074 PMC4751570

[8] Badoud D, Tsakiris M. From the body’s viscera to the body’s image: Is there a link between interoception and body image concerns? Neurosci Biobehav Rev. 2017;77:237–46. 28377099 10.1016/j.neubiorev.2017.03.017

[9] Emanuelsen L, Drew R, Köteles F. Interoceptive sensitivity, body image dissatisfaction, and body awareness in healthy individuals. Scand J Psychol. 2015;56(2):167–74. 25444023 10.1111/sjop.12183

[10] Todd J, et al. Multiple dimensions of interoceptive awareness are associated with facets of body image in british adults. Body Image. 2019;29:6–16. 30771695 10.1016/j.bodyim.2019.02.003

[11] Atanasova K, Lotter T, Reindl W, Lis S. Multidimensional assessment of interoceptive abilities, emotion processing and the role of early life stress in inflammatory bowel diseases. Front Psych. 2021;12:680878. 10.3389/fpsyt.2021.680878. 10.3389/fpsyt.2021.680878 PMC8264143 34248716

[12] Labus JS, et al. The central role of gastrointestinal-specific anxiety in irritable bowel syndrome: further validation of the visceral sensitivity index. Psychosom Med. 2007;69(1):89–98. 17244851 10.1097/PSY.0b013e31802e2f24

[13] Suzuki K, et al. Multisensory integration across exteroceptive and interoceptive domains modulates self-experience in the rubber-hand illusion. Neuropsychologia. 2013;51(13):2909–17. 23993906 10.1016/j.neuropsychologia.2013.08.014

[14] Tsakiris M, Jiménez AT, Costantini M. Just a heartbeat away from one's body: interoceptive sensitivity predicts malleability of body-representations. Proc R Soc B: Biol Sci. 2011;278(1717):2470-2476. 10.1098/rspb.2010.2547 PMC3125630 21208964

[15] Crucianelli L, et al. Interoceptive ingredients of body ownership: ffective touch and cardiac awareness in the rubber hand illusion. Cortex. 2018;104:180–92. 28532579 10.1016/j.cortex.2017.04.018

[16] Srinath A, Young E, Szigethy E. Pain management in patients with inflammatory bowel disease: translational approaches from bench to bedside. Inflamm Bowel Dis. 2014;20(12):2433–49. 25208108 10.1097/MIB.0000000000000170

[17] Cordier L, et al. Synchronous stimulation with light and heat induces body ownership and reduces pain perception. J Pain. 2020;21(5):700–7. 31698132 10.1016/j.jpain.2019.10.009

[18] Solcà M, et al. Behavioral and neurophysiological evidence for altered interoceptive bodily processing in chronic pain. Neuroimage. 2020;217: 116902. 32438047 10.1016/j.neuroimage.2020.116902

[19] Lewis JS, et al. Body perception disturbance: a contribution to pain in complex regional pain syndrome (CRPS). PAIN®. 2007;133(1–3):111–9. 17509761 10.1016/j.pain.2007.03.013

[20] Lewis JS, et al. Visual illusions modulate body perception disturbance and pain in Complex Regional Pain Syndrome: A randomized trial. Eur J Pain. 2021;25(7):1551–63. 33759278 10.1002/ejp.1766

[21] Moseley GL, Gallace A, Iannetti GD. Spatially defined modulation of skin temperature and hand ownership of both hands in patients with unilateral complex regional pain syndrome. Brain. 2012;135(12):3676–86. 23250885 10.1093/brain/aws297

[22] Markey CH, Dunaev JL, August KJ. Body image experiences in the context of chronic pain: An examination of associations among perceptions of pain, body dissatisfaction, and positive body image. Body Image. 2020;32:103–10. 31862524 10.1016/j.bodyim.2019.11.005

[23] Hassani F, Koraei A, Yaghoobi R, Zarea K. An evaluating of the relationship between body image, body satisfaction, depression, marital quality, and self-esteem in patients with psoriasis. Psychol Health Med. 2020;26(4):467–77. 10.1080/13548506.2020.1766093. 10.1080/13548506.2020.1766093 32426997

[24] Scheffers M, et al. Body image in patients with mental disorders: Characteristics, associations with diagnosis and treatment outcome. Compr Psychiatry. 2017;74:53–60. 28095340 10.1016/j.comppsych.2017.01.004

[25] Löffler A, et al. Reductions in whole-body ownership in borderline personality disorder - a phenomenological manifestation of dissociation. J Trauma Dissociation. 2020;21(2):264–77. 31646957 10.1080/15299732.2019.1678213

[26] Dyer A, et al. Body image in patients with posttraumatic stress disorder after childhood sexual abuse and co-occurring eating disorder. Psychopathology. 2013;46(3):186–91. 22964627 10.1159/000341590

[27] Lanius RA, et al. Emotion modulation in PTSD: linical and neurobiological evidence for a dissociative subtype. Am J Psychiatry. 2010;167(6):640–7. 20360318 10.1176/appi.ajp.2009.09081168 PMC3226703

[28] Courtois CA, Ford JD. Treating complex traumatic stress disorders: n evidence-based guide. New York: Guilford Press; 2009.

[29] Van der Kolk BA, et al. Disorders of extreme stress: the empirical foundation of a complex adaptation to trauma. J Trauma Stress: Off Publ Int Soc Trauma Stress Stud. 2005;18(5):389–99. 10.1002/jts.20047 16281237

[30] Van der Kolk B. The body keeps the score: mind, brain and body in the transformation of trauma. UK: Penguin; 2014.

[31] Herzog JI, Schmahl C. Adverse childhood experiences and the consequences on neurobiological, psychosocial, and somatic conditions across the lifespan. Front Psychiatry. 2018;9: 420. 30233435 10.3389/fpsyt.2018.00420 PMC6131660

[32] Kleindienst N, et al. Evaluation of the own body in women with current and remitted borderline personality disorder: evidence for long-lasting effects of childhood sexual abuse. Eur J Psychotraumatol. 2020;11(1): 1764707. 33029307 10.1080/20008198.2020.1764707 PMC7473052

[33] Scheffers M, et al. Negative body experience in women with early childhood trauma: associations with trauma severity and dissociation. Eur J Psychotraumatol. 2017;8(1): 1322892. 28649300 10.1080/20008198.2017.1322892 PMC5475325

[34] Straus MB. Abuse and victimization across the life span. Baltimore: Johns Hopkins University Press; 1988.

[35] Dyer A, et al. Body image disturbance in patients with borderline personality disorder: Impact of eating disorders and perceived childhood sexual abuse. Body Image. 2013;10(2):220–5. 23375838 10.1016/j.bodyim.2012.12.007

[36] Bekrater-Bodmann R, et al. Body plasticity in borderline personality disorder: A link to dissociation. Compr Psychiatry. 2016;69:36–44. 27423343 10.1016/j.comppsych.2016.05.002

[37] Sack M, Boroske-Leiner K, Lahmann C. Association of nonsexual and sexual traumatizations with body image and psychosomatic symptoms in psychosomatic outpatients. Gen Hosp Psychiatry. 2010;32(3):315–20. 20430236 10.1016/j.genhosppsych.2010.01.002

[38] Price C. Body-oriented therapy in recovery from child sexual abuse: an efficacy study. Altern Ther Health Med. 2005;11(5):46. 16189948 PMC1933482

[39] Walker HE, Wamser-Nanney R. Revictimization risk factors following childhood maltreatment: A literature review. Trauma Violence Abuse. 2023;24(4):2319–32. 35476548 10.1177/15248380221093692

[40] Tsur N, et al. The traumatized body: Long-term PTSD and its implications for the orientation towards bodily signals. Psychiatry Res. 2018;261:281–9. 29329049 10.1016/j.psychres.2017.12.083

[41] Danese A, Lewis SJ. Psychoneuroimmunology of early-life stress: the hidden wounds of childhood trauma? Neuropsychopharmacology. 2017;42(1):99–114. 27629365 10.1038/npp.2016.198 PMC5143500

[42] Baumeister D, et al. Childhood trauma and adulthood inflammation: a meta-analysis of peripheral C-reactive protein, interleukin-6 and tumour necrosis factor-α. Mol Psychiatry. 2016;21(5):642–9. 26033244 10.1038/mp.2015.67 PMC4564950

[43] Witges KM, et al. The relationship between adverse childhood experiences and health care use in the Manitoba IBD cohort study. Inflamm Bowel Dis. 2019;25(10):1700–10. 30919910 10.1093/ibd/izz054 PMC6749885

[44] Wan A, et al. Childhood maltreatment and psychiatric comorbidity in immune-mediated inflammatory disorders. Psychosom Med. 2022;84(1):10–9. 34654023 10.1097/PSY.0000000000001025

[45] Schaan VK, et al. Childhood trauma affects stress-related interoceptive accuracy. Front Psychiatry. 2019;10: 750. 31681049 10.3389/fpsyt.2019.00750 PMC6813623

[46] Faul F, et al. G*Power 3: a flexible statistical power analysis program for the social, behavioral, and biomedical sciences. Behav Res Methods. 2007;39(2):175–91. 17695343 10.3758/bf03193146

[47] Bernstein CN, et al. World Gastroenterology Organization Practice Guidelines for the diagnosis and management of IBD in 2010. Inflamm Bowel Dis. 2010;16(1):112–24. 19653289 10.1002/ibd.21048

[48] Gomollón F, et al. 3rd European evidence-based consensus on the diagnosis and management of Crohn’s disease 2016: part 1: diagnosis and medical management. J Crohns Colitis. 2017;11(1):3–25. 27660341 10.1093/ecco-jcc/jjw168

[49] Margo F, et al. Third european evidence-based consensus on diagnosis and management of ulcerative colitis. Part1: definitions, diagnosis, extra-intestinal manifestations, pregnancy, cancer surveillance, surgery, and ileo-anal pouch disorders. J Crohns Colitis. 2017;11(6):648–70. 10.1093/ecco-jcc/jjx008 28158501

[50] Annese V, et al. European evidence based consensus for endoscopy in inflammatory bowel disease. J Crohns Colitis. 2013;7(12):982–1018. 24184171 10.1016/j.crohns.2013.09.016

[51] Margraf J, Cwik JC. Mini-DIPS open access. Aufl. 2017;7:590–2.

[52] Labus JS, et al. The Visceral Sensitivity Index: development and validation of a gastrointestinal symptom-specific anxiety scale. Aliment Pharmacol Ther. 2004;20(1):89–97. 15225175 10.1111/j.1365-2036.2004.02007.x

[53] Mehling WE, et al. The multidimensional assessment of interoceptive awareness (MAIA). PLoS ONE. 2012;7(11): e48230. 23133619 10.1371/journal.pone.0048230 PMC3486814

[54] Rosenberg M. Rosenberg self-esteem scale (RSE). Acceptance Commitment Ther Measures Packag. 1965;61(52):18.

[55] Klinitzke G, et al. The German Version of the Childhood Trauma Questionnaire (CTQ): psychometric characteristics in a representative sample of the general population. Psychother Psychosom Med Psychol. 2012;62(2):47–51. 22203470 10.1055/s-0031-1295495

[56] Weathers FW, et al. The ptsd checklist for dsm-5 (pcl-5). Scale available from the National Center for PTSD at www.ptsd.va.gov, 2013;10. Accessed Jan 2019.

[57] Nummenmaa L, et al. Bodily maps of emotions. Proc Natl Acad Sci U S A. 2014;111(2):646–51. 24379370 10.1073/pnas.1321664111 PMC3896150

[58] Hayes AF. Introduction to mediation, moderation, and conditional process analysis: a regression-based approach. Guilford Publications; 2017.

[59] Benjamini Y, Hochberg Y. Controlling the false discovery rate: a practical and powerful approach to multiple testing. J Roy Stat Soc: Ser B (Methodol). 1995;57(1):289–300.

[60] Beese SE, et al. Body image dissatisfaction in patients with inflammatory bowel disease: a systematic review. BMJ Open Gastroenterol. 2019;6(1): e000255. 30899537 10.1136/bmjgast-2018-000255 PMC6398870

[61] Matamala-Gomez M, et al. Changing body representation through full body ownership illusions might foster motor rehabilitation outcome in patients with stroke. Front Psychol. 2020;11:1962. 10.3389/fpsyg.2020.01962 PMC7471722 32973612

[62] Botvinick M, Cohen J. Rubber hands ‘feel’ touch that eyes see. Nature. 1998;391(6669):756–756. 9486643 10.1038/35784

[63] Ceuleers H, et al. Visceral hypersensitivity in inflammatory bowel diseases and irritable bowel syndrome: The role of proteases. World J Gastroenterol. 2016;22(47):10275–86. 28058009 10.3748/wjg.v22.i47.10275 PMC5175241

[64] Bultitude JH, Rafal RD. Derangement of body representation in complex regional pain syndrome: report of a case treated with mirror and prisms. Exp Brain Res. 2010;204(3):409–18. 19967390 10.1007/s00221-009-2107-8 PMC2895899

[65] Brooks J, Tracey I. The insula: a multidimensional integration site for pain. Pain. 2007;128:1–2. 10.1016/j.pain.2006.12.025 17254713

[66] Tsakiris M, et al. Neural signatures of body ownership: a sensory network for bodily self-consciousness. Cereb Cortex. 2006;17(10):2235–44. 17138596 10.1093/cercor/bhl131

[67] Hong J-Y, et al. Regional neuroplastic brain changes in patients with chronic inflammatory and non-inflammatory visceral pain. PLoS ONE. 2014;9(1): e84564. 24416245 10.1371/journal.pone.0084564 PMC3885578

[68] Thomann AK, et al. Altered markers of brain development in Crohn’s Disease with extraintestinal manifestations - a pilot study. PLoS ONE. 2016;11(9): e0163202. 27655165 10.1371/journal.pone.0163202 PMC5031401

[69] Hood MM, Jedel S. Mindfulness-based interventions in inflammatory bowel disease. Gastroenterol Clin North Am. 2017;46(4):859–74. 29173527 10.1016/j.gtc.2017.08.008

[70] Shah K, et al. Mind-body treatments of irritable bowel syndrome symptoms: An updated meta-analysis. Behav Res Ther. 2020;128: 103462. 32229334 10.1016/j.brat.2019.103462

[71] Ewais T, et al. A systematic review and meta-analysis of mindfulness based interventions and yoga in inflammatory bowel disease. J Psychosom Res. 2019;116:44–53. 30654993 10.1016/j.jpsychores.2018.11.010

[72] Kearney DJ, et al. Association of participation in a mindfulness programme with bowel symptoms, gastrointestinal symptom-specific anxiety and quality of life. Aliment Pharmacol Ther. 2011;34(3):363–73. 21651595 10.1111/j.1365-2036.2011.04731.x

[73] Bekrater-Bodmann R, et al. Interoceptive awareness is negatively related to the exteroceptive manipulation of bodily self-location. Front Psychol. 2020;11:562016–562016. 33343444 10.3389/fpsyg.2020.562016 PMC7746809

[74] Schroter FA, Siebertz M, Jansen P. The impact of a short body-focused meditation on body ownership and interoceptive abilities. Mindfulness. 2023;14(1):159–73.

[75] Cruz-Sáez S, et al. The effect of body dissatisfaction on disordered eating: The mediating role of self-esteem and negative affect in male and female adolescents. J Health Psychol. 2020;25(8):1098–108. 30101609 10.1177/1359105317748734

[76] Murphy J, Catmur C, Bird G. Classifying individual differences in interoception: Implications for the measurement of interoceptive awareness. Psychon Bull Rev. 2019;26(5):1467–71. 31270764 10.3758/s13423-019-01632-7 PMC6797703

