# Supplementary material for: Body Evaluation and Body Ownership in Patients with Inflammatory Bowel Disease: the Role of Interoceptive Sensibility and Childhood Maltreatment
Source: Int J Behav Med. 2024 Aug 21;32(6):821–33. doi: 10.1007/s12529-024-10316-z (PMC12847169; doi:10.1007/s12529-024-10316-z)
Supplement: Supplementary file 1 — Supplementary file1 (DOCX 676 KB) [file 12529_2024_10316_MOESM1_ESM.docx]

## **Body evaluation and body ownership in patients with inflammatory bowel disease: the role of interoceptive sensibility and adverse childhood experiences**

**Supplemental Digital Content**

**Table S1.** Treatment medication reported in the IBD group (N=41)

| **IBD medication** | **N** |
| --- | --- |
| Biologics | 40 |
| Mesalazine | 1 |

**Table S2.** Histories of further somatic conditions in the IBD group (whole IBD sample: N=41, patients reporting further somatic diseases: N=10)

| **Somatic condition** | **N** |
| --- | --- |
| Pulmonary embolism | 2 |
| Pancreatitis | 2 |
| Diabetes | 1 |
| Chronic cystitis | 1 |
| Thrombocythemia | 1 |
| Asthma bronchiale | 1 |
| Hypothyoroidism | 2 |

**
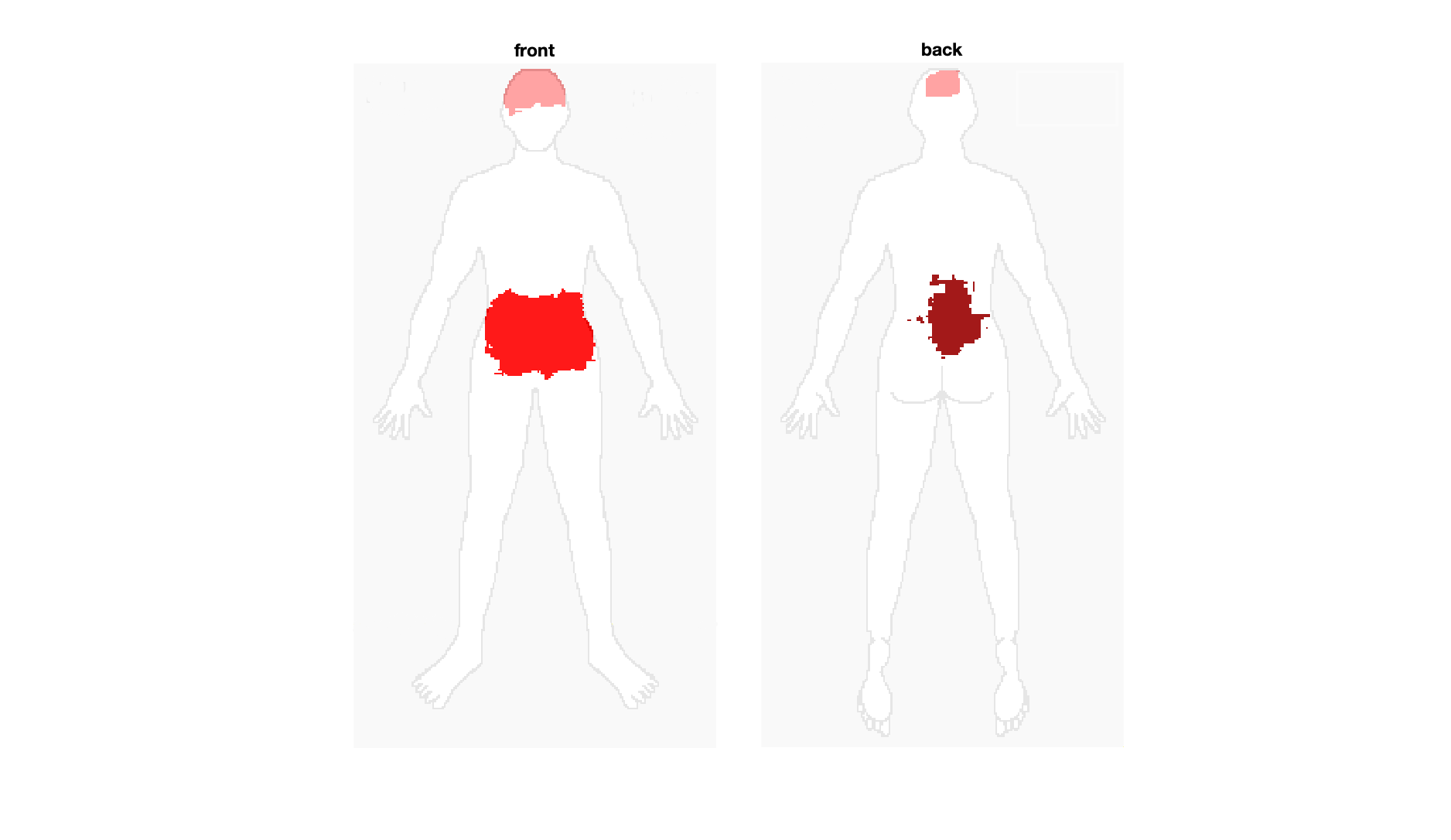
**

**Figure S1.** Pain-related ROI with mean pain intensity ≥ 1.60 computed across all participants (N=85). Colored body areas (head, abdomen, back) were used as pre-defined ROI for further analyses. Disease-related pain ROI includes the abdomen (red area), disease-unrelated pain ROIs include the head (pink area) and the back (dark red area).


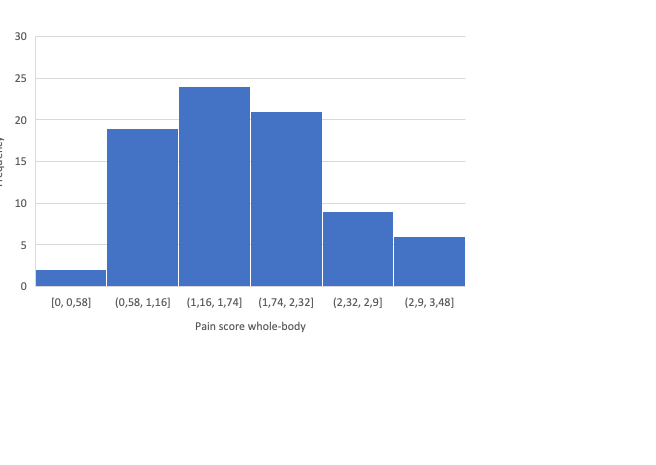


**Figure S2.** Distribution of whole-body scores for pain experience during the last four weeks prior the experiment. Scores were computed across n=85 participants (M = 1.61; SD = 0.74).

**Table S3.** Number of coloured pixels within the whole-body template and the pre-defined ROI.

|  | IBD | HC | p-value |
| --- | --- | --- | --- |
|  | N pixels | N pixels | *p* |
| *Body satisfaction* | | | |
| Whole-body | 26382.3 | 28022.3 | .717 |
| Head | 289.3 | 223.4 | .066 |
| Abdomen | 1678.6 | 1687.0 | .922 |
| Back | 514.4 | 541.8 | .767 |
| *Body ownership* | | | |
| Whole-body | 33608.3 | 34172.6 | . 201 |
| Head | 320.6 | 315.0 | .880 |
| Abdomen | 1807.8 | 1742.3 | .789 |
| Back | 678.8 | 687.3 | .689 |

Note: group comparisons were computed using Mann-Whitney U tests.

**Table S4.** Spearman rank correlations (r_s_) between Body image scores, BMI, number of IBD-related surgeries in the past and IBD symptoms severity.

|  | BMI | | Number of surgeries | | IBD severity | | |
| --- | --- | --- | --- | --- | --- | --- | --- |
|  | *r_s_* | *p* | *r_s_* | *p* | *r_s_* | *p* |  |
| *Body satisfaction* | | | | | | | |
| Whole-body | -.202 | .217 | -.021 | .897 | -.072 | .709 |  |
| ROI Abdomen | -.260 | .131 | -.010 | .953 | -.154 | .443 |  |
| ROI Head + Back | -.034 | .848 | -.129 | .459 | -.138 | .501 |  |
| *Body ownership* | | | | | | | |
| Whole-body | -.220 | .190 | -.028 | .870 | -.119 | .554 |  |
| ROI Abdomen | -.182 | .282 | .123 | .469 | -.041 | .837 |  |
| ROI Head + Back | -.226 | .184 | .082 | .636 | -.001 | .996 |  |

Note: correlations were computed only in the IBD group (n=41). IBD, inflammatory bowel disease group; HC, healthy control group; BMI, body mass index; ROI, region of interest.


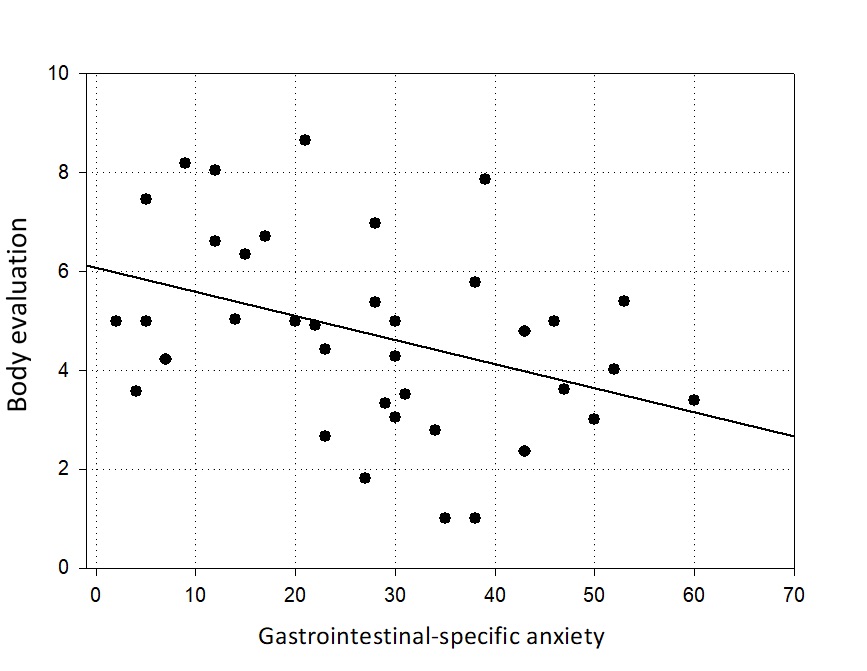


**Figure S3.** Association between gastrointestinal-specific anxiety (VSI total score) and body evaluation of the abdomen (0 = “very negative”; 9 = “very positive”) among IBD patients. Participants reporting higher gastrointestinal-specific anxiety rated their abdomen as less positive, indicating higher body dissatisfaction with this pain-associated body area.


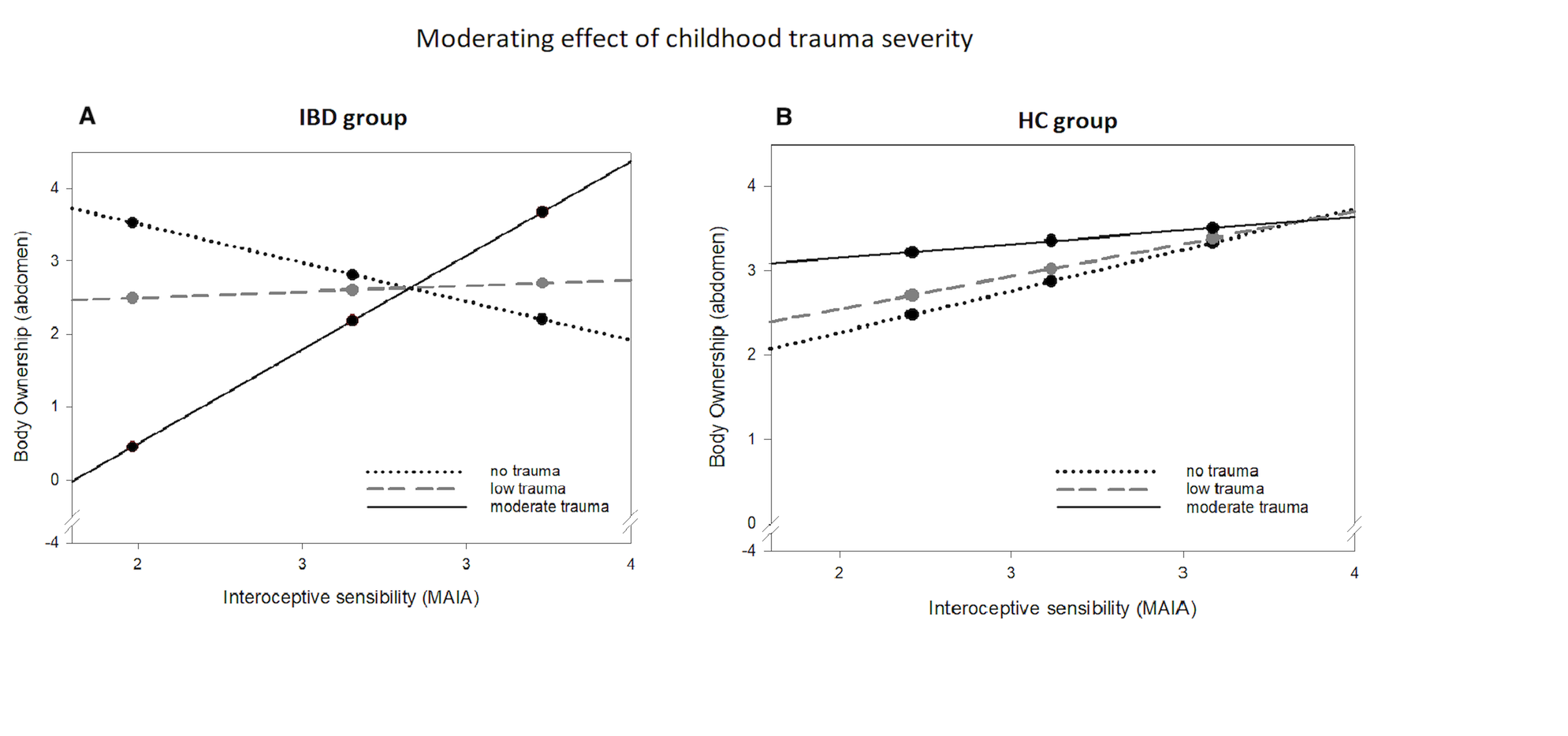


**Figure S4.** Moderation effect of adverse childhood experiences (CTQ total score) on the link between interoceptive sensibility (MAIA total score) and body ownership of the abdomen (- 4= “low ownership”; 4= “high ownership”) in IBD (Fig. S4 A) and HC participants (Fig. S4 B). The interaction between ACE and interoceptive sensibility was significant only in the IBD group and only for patients with moderate CTQ scores (solid black line in Fig. S4 A).
